# Genetic Association of Primary Lung Cancer With Urological Cancers: A Bidirectional Mendelian Randomization Study and SEER Database Validation

**DOI:** 10.1002/cam4.71272

**Published:** 2025-09-29

**Authors:** Hao Zhou, Zihao Feng, Cunjing Zheng, Ling Xia, Ziran Dai, Zheyu Ai, Zhenwei Li, Kezhi Liu, Yinghan Wang, Ning Su, Zhenhua Chen, Jing Zhang, Xiaohan Jin

**Affiliations:** ^1^ State Key Laboratory of Respiratory Disease, Guangzhou Medical University, Institute of Tuberculosis, Institute of Pulmonary Diseases, Department of Oncology Guangzhou Chest Hospital Affiliated to Guangdong Pharmaceutical University Guangzhou China; ^2^ Department of Urology The First Affiliated Hospital, Sun Yat‐sen University Guangzhou China; ^3^ Department of Radiology Sun Yat‐sen Memorial Hospital, Sun Yat‐sen University Guangzhou China; ^4^ Department of Cataract, Zhongshan Ophthalmic Center Sun Yat‐sen University Guangzhou China; ^5^ Department of Pulmonary and Critical Care Medicine The First Affiliated Hospital, Sun Yat‐sen University Guangzhou China

**Keywords:** GRS, lung cancer, Mendelian randomization, SEER database, urological cancer

## Abstract

**Background:**

Previous studies reported an association between lung cancer (LC) and partial urological cancers (UCs). However, the exact causal association between LC and UCs remains obscure.

**Methods:**

A two‐sample bidirectional Mendelian randomization (MR) and the Genetic Risk Scores (GRS) method were used to assess the genetic relationships between LC and UCs. The risk of second primary cancer (SPC) was validated using the Surveillance, Epidemiology, and End Results (SEER) database.

**Results:**

MR analysis demonstrated genetic associations between overall LC and lung adenocarcinoma (LUAD) with renal cell carcinoma (RCC) (overall LC: OR [95% CI] = 1.214 [1.003–1.469], *p* = 0.046; LUAD: OR [95% CI] = 1.144 [1.029–1.271], *p* = 0.012). The GRS method also yielded consistent results (overall LC: OR [95% CI] = 1.229 [1.067–1.414], *p* = 0.004; LUAD: OR [95% CI] = 1.125 [1.019–1.243], *p* = 0.020). Therefore, this study primarily focused on the significant associations between overall LC and LUAD with RCC. Meanwhile, the SEER database was exploited to confirm the correlation between primary LUAD (PLUAD) and secondary primary RCC (SPC‐RCC). The results indicated that the risk of SPC‐RCC after PLUAD was substantially higher than the US reference population.

**Conclusions:**

The MR study identified genetic associations between LC and UCs, revealing an elevated risk of SPC‐RCC after primary LC (PLC), particularly LUAD. This study lays a foundation for SPC‐RCC prevention after PLC, indicating the necessity of enhanced surveillance of PLC patients in clinical practice and further research into the shared biological pathways to provide innovative therapeutic alternatives.

**Abbreviations**
BCa: bladder cancer
CI: confidence interval
EAR: excess absolute risk
GRS: genetic risk score
GWAS: genome‐wide association study
InSIDE: instrument strength independent of direct effect
IVs: instrumental variables
IVW: inverse variance weighted
LC: lung cancer
LD: linkage disequilibrium
LUAD: lung adenocarcinoma
LUSC: squamous cell lung carcinoma
MP‐SIR: multiple primary standardized incidence ratios
MR: Mendelian randomization
MR‐PRESSO: Mendelian randomization pleiotropy residual sum and outlier
MR‐RAPS: MR‐robust adjusted profile score
NSCLC: non‐small cell lung cancer
PCa: prostate cancer
PLC: primary lung cancer
PLUAD: primary lung adenocarcinoma
RCC: renal cell carcinoma
SCLC: small cell lung cancer
SEER: Surveillance, Epidemiology and End Results
SNP: single nucleotide polymorphism
SPC: second primary cancer
SPC‐RCC: second primary RCC
UCs: urological cancers
WM: weighted median

## 1 Introduction

Globally, lung cancer (LC) has been the leading factor of cancer‐associated deaths, with both incidence and mortality rates rising over recent decades [1]. Despite advancements in treatment, the prognosis for patients with the late phase of LC remains unfavorable, distinguished by a markedly low 5‐year survival rate. It is classified into non‐small cell lung cancer (NSCLC) and small cell lung cancer (SCLC), with NSCLC representing roughly 85% of all cases [2]. Moreover, NSCLC comprises multiple cancer subtypes, with lung adenocarcinoma (LUAD) comprising the predominant categories. LUAD represents the predominant variant of NSCLC and accounts for approximately 40% of LCs [2]. Some of the risk factors associated with LC are smoking, environmental pollution, obesity, and genetic predispositions [3, 4]. These factors have a strong association with urological cancers (UCs) [5, 6], specifically renal cell carcinoma (RCC), bladder cancer (BCa), and prostate cancer (PCa). Lung cancer and UCs rank among the most prevalent malignancies globally. Previous studies have indicated a possible correlation between LC and UCs, particularly PCa and BCa [7]. However, most studies to date have been observational, restricting the capacity to establish causal relationships. Therefore, there is an urgent need for more rigorous methodologies to explore potential causal relationships between LC and UCs, which may yield significant insights for therapeutic management.

In genetic epidemiology, Mendelian randomization (MR) is a widely utilized method for etiological inference [8]. Based on Mendel's laws, parental alleles are randomly assigned to progeny during the formation of gametes in the MR method, which is identical to a randomized controlled trial (RCT) design [9]. The continual development of MR methods has rendered it an effective method for gene‐level studies that aim to infer pathogenic correlations between complicated disorders. For instance, Xian et al. [10] conducted a two‐sample MR analysis to examine the relationship between Graves disease and inflammatory bowel disease as per a genome‐wide association study (GWAS) with a large sample. Further, Liu et al. [11] used MR to explore the correlation between gastroesophageal reflux disease and LC. Their findings indicated that gastroesophageal reflux disease was related to an elevated incidence of LC.

This study mainly examines the causal relationship between LC and its subtypes and UCs using MR, with validation conducted through the Surveillance, Epidemiology and End Results (SEER) database. This comprehensive analysis of potential connections between these cancers provides insights into shared and distinct etiological factors that could enhance prevention and treatment strategies. Further, extending the comprehension of the underlying etiological mechanisms through validation with SEER population‐based data can potentially guide future cancer prevention and treatment initiatives.

## 2 Methods

### 2.1 Study Design

**FIGURE 1:**
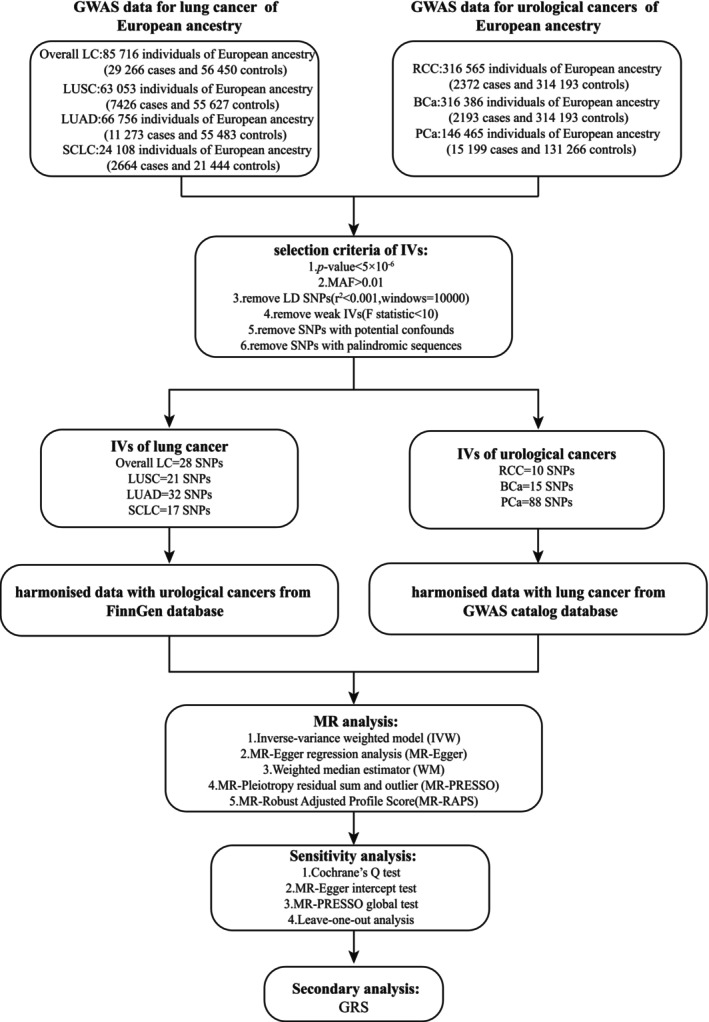
Study design and overview of Mendelian randomization (MR) study. BCa, bladder cancer; GRS, genetic risk score; GWAS, genome‐wide association study; IVs, instrumental variables; LC, lung cancer; LD, linkage disequilibrium; LUAD, lung adenocarcinoma; LUSC, squamous cell lung carcinoma; MAF, minor allele frequency; PCa, prostate cancer; RCC, renal cell carcinoma; SCLC, small cell lung cancer; SNP, single nucleotide polymorphism.

Figure 1 depicts the overview of the MR study design. All instrumental variables (IVs) were selected as per the three basic assumptions of MR analyses: (1) the genetic variant has a robust correlation with the exposure; (2) the genetic variant is independent of confounders that might affect the exposure–outcome interaction; and (3) the genetic variant affects the outcome solely through the exposure [9, 12].

### 2.2 Data Sources

Summary‐level data from publicly accessible GWAS was used for MR analysis. The study sample was restricted to a specific European population. Summary statistics for LC were obtained from the GWAS Catalog (https://www.ebi.ac.uk/gwas/home). Moreover, the data for LC were retrieved from a large‐scale GWAS study that involved 85,716 participants (cases 29,266 and controls 56,450). The GWAS study also classified LC into specific pathological types, including LUAD, LUSC, and SCLC [13]. To avoid sample overlap as much as possible, summary statistics for UCs were collected from the FinnGen database (https://www.finngen.fi/en). These UCs included RCC, BCa, and PCa. The summary‐level GWAS data for RCC (finn‐R10‐C3_KIDNEY_NOTRENALPELVIS_EXALLC) comprised 2372 cases and 314,193 controls. The BCa dataset (finngen_R10_C3_BLADDER_EXALLC) comprised 2193 cases and 314,193 controls. The PCa dataset (finngen_R10_C3_PROSTATE_EXALLC) had 15,199 cases and 131,266 controls. The original GWAS received permission from their respective institutions, and all data used for this analysis was publicly accessible. Thus, no further ethical approval was needed.

### 2.3 Selection of IVs

Genetic IVs for LC and UCs were constructed based on the following criteria: (a) considering the limited availability of accessible single nucleotide polymorphisms (SNPs), SNPs were selected with a threshold of *p* < 5e^−6^; (b) to reduce linkage disequilibrium (LD), a clumping algorithm was used with parameters of *r* ^2^ = 0.001 and kb = 10,000, as genetic variants in close proximity on the genome were often inherited together, a phenomenon referred to as LD; (c) minor allele frequency (MAF) > 0.01 (mutations were present in > 1% of the population); (d) the statistical strength of each SNP was evaluated by excluding SNPs with *F* statistic < 10 for weak IVs [14]; (e) to reduce the influence of confounding factors in the MR analysis, IVs correlated with confounders (smoking and BMI) were excluded using the LDtrait Tool (https://ldlink.nih.gov/?tab=ldtrait) and the IEU database (https://gwas.mrcieu.ac.uk/). Due to the unavailability of PhenoScanner, the LDtrait tool was used to find relevant confounders. The IEU database identified the traits of SNPs not found in LDtrait; (f) palindromic SNPs were excluded during the harmonization phase; these refer to SNPs where the base sequence is identical on both the forward and reverse DNA strands but in opposite orientations; and (g) if the Mendelian randomization pleiotropy residual sum and outlier (MR‐PRESSO) method detected any outlier SNPs, those outliers were excluded, and the remaining IVs were statistically analyzed.

### 2.4 MR Analysis

The main strategy employed in this study was the inverse variance weighted (IVW) method to evaluate the bidirectional causal relationship between LC and UCs for the MR analyses. Based on the meta‐analysis, the IVW method was the most prevalent and extensively employed for MR analysis. It combines ratio estimates of SNPs in an IVW manner to determine the effect of risk factors on outcomes [15]. Both random‐effects and fixed‐effects models were included in the IVW method. When heterogeneity was detected in the MR analysis, the random‐effects IVW was employed, as it resisted biases from weaker SNP‐exposure associations [16]. Moreover, the weighted median (WM), MR‐Egger, MR‐Pleiotropy Residual Sum and Outlier (MR‐PRESSO), and MR‐Robust Adjusted Profile Score (MR‐RAPS) methods were used to examine whether LC and UCs were genetically correlated. A significance level of *p* < 0.05 was regarded as statistically significant. The WM method was highly robust to pleiotropic genetic variation, resulting in relatively consistent effect estimates even when up to half of the IVs were invalid [17]. The MR‐Egger method assessed whether genetic variants showed average pleiotropic effects on the outcome that deviated from zero (directional pleiotropy) and presented an accurate indication of the causal effect under the weaker Instrument Strength Independent of Direct Effect (InSIDE) assumption [18]. Under the horizontal pleiotropy and InSIDE assumptions, MR‐PRESSO assumed that a minimum of 50% of the genetic variants were valid. It served three primary functions: (1) the “MR‐PRESSO global test”, which assessed the horizontal pleiotropy extent; (2) the “MR‐PRESSO outlier test”, which excluded abnormal genetic variants (outliers) and provided corrected estimates; and (3) the “MR‐PRESSO distortion test”, which evaluated differences between pre‐corrected and corrected outcomes [17]. The MR‐RAPS with a Huber loss function could mimic the random‐effects distribution of pleiotropic effects. MR‐RAPS proved highly effective in numerical patterns when systematic and idiosyncratic pleiotropy was considered. It is highly suggested that it is a reliable tool for routine MR analysis, specifically when addressing intricate traits that involve exposure and outcome [19].

### 2.5 Sensitivity Analysis

Given that the IVW method could be biased by pleiotropic and heterogeneous IVs, sensitivity analyses were conducted to reduce these effects in the causal estimates. Cochrane's *Q* test was used to examine potential heterogeneity. In cases where heterogeneity was detected (*p* < 0.05), a random‐effects IVW analysis was performed to account for the observed heterogeneity. Further, the intercept of MR‐Egger and MR‐PRESSO global tests were implemented to determine the existence of horizontal pleiotropy among the genetic variants (*p* < 0.05 represented potential horizontal pleiotropy).

### 2.6 Genetic Risk Scores (GRSs)

A secondary analysis was conducted using the GRS approach to confirm the above mentioned MR results. The study also performed the analyses via R (v 4.3.2) with the “gtx” R package, which includes the GRS function within its grs.summary module. This module used single SNP association summary data from GWAS results, similar to a method that regressed an outcome onto an additive GRS [17, 20, 21]. For uncorrelated SNPs, the causal estimate (*α*) can be calculated as $\alpha ≅\frac{\sum \omega \beta {\mathrm{Se}}_{\beta }^{-2}}{\sum \omega^{2}{\mathrm{Se}}_{\beta }^{-2}}$, with its standard error (se*_α_*) estimated as ${\mathrm{Se}}_{\alpha }≅\frac{1}{\sum^{\omega^{2}}{\mathrm{Se}}_{\beta }^{-2}}$. In this context, ω represents the estimated effects on the intermediate trait or biomarker, while *β* corresponds to the estimated effects on the response variable or outcome with standard errors se*_β_*. A detailed description of the method can be found elsewhere [22]. Furthermore, previous studies have shown that this MR method, which uses meta‐GWAS summary data, is just as efficient as the one that uses individual‐level data [23].

A two‐sided statistical analysis was performed, with a significance threshold at *p* < 0.05. All analyses were carried out using R v 4.3.2 and the packages “MendelianRandomization,” “TwosampleMR,” “RAPS,” “PRESSO” and “gtx.”

### 2.7 Power Analysis

Statistical power was assessed using the online tool mRnd (https://shiny.cnsgenomics.com/mRnd/) to estimate the probability of correctly detecting causal effects [14].

### 2.8 SEER Database and Statistical Analysis

The SEER database is an essential resource for assessing the risks of second primary cancers (SPCs) due to its large sample size, covering approximately 47.9% of the US population, and extensive follow‐up of cancer survivors. This study retrospectively recruited patients with LUAD, RCC, and second primary RCC (SPC‐RCC) after primary LUAD (PLUAD) from the SEER database and obtained the relevant clinicopathological data. The inclusion criteria were: (a) diagnosed from 2004 to 2015; (b) aged > 18 and < 90 years; (c) white race; and (d) diagnosis histologically confirmed as LUAD and RCC. The exclusion criteria were: (a) cases in which the second malignant tumor has the same histology as the first malignant tumor; (b) patients with unknown TNM staging (Tx, Nx, Mx) as per the 6th edition of the American Joint Committee on Cancer stage system; (c) the type of reporting source was “Autopsy only” or “Death certificate only”; (d) patients diagnosed with a SPC within 6 months of the onset of the first primary malignancy; and (e) incomplete information.

The multiple primary standardized incidence ratios (MP‐SIR) were defined as the ratio of the observed incidence of a second cancer in patients previously diagnosed with a specific type of cancer to the expected incidence derived from the SEER reference population. All standardized incidence ratios (SIR) and their related 95% confidence intervals (95% CI) were obtained using the “MP‐SIR” session of SEER*Stat (v 8.4.3) [24]. The MP‐SIR session was used to determine whether the occurrence of observed cases with SPCs was greater or less than anticipated at random in a comparable US population with similar age and gender distributions during the same follow‐up period. The ratio of observed to expected (O/E) SPCs, also referred to as the SIR or the relative risk ratio, was determined by considering that the cancer incidence rates of the sample and the reference population were comparable [25]. The extracted SEER*Stat tables were marked with a red hash (#) to indicate that the estimated SIRs with 95% CI that did not include “1” were statistically significant. Excess absolute risk (EAR) was calculated to quantify the burden of SPCs in the specified population. EAR was characterized as the difference between the observed and the expected cases with SPCs, divided by the number of person‐years‐at‐risk (PYR) observed. The formula for calculating EARs was expressed as [(O‐E)/PYR] × 10,000 and expressed in terms of 10,000 PYR [26]. The patients who suffered from primary LUAD followed by primary RCC between 1975 and 2021 were selected. The study was limited to the white population, with a minimum latency period of 6 months between the first and second primary diagnoses [27]. All statistical analyses were conducted with SEER*STAT and R software (v 4.3.2). A two‐tailed *p*‐value of less than 0.05 was regarded as significant.

## 3 Results

### 3.1 Selection and Validation of IVs

The selection procedure for IVs in this study is illustrated in Figure 1. Approximately 58 overall LC, 43 LUAD, 38 LUSC, and 25 SCLC SNPs that satisfied the criteria of independence from LD, *p* < 5 × 10^−6^, and MAF > 0.01 were enrolled as IVs. All selected SNPs had an *F* statistic greater than 10, indicating a lack of weak instrument bias. The study removed IVs associated with confounders (smoking and BMI) and palindromic sequences (30 overall LC, 11 LUAD, 17 LUSC, and 8 SCLC SNPs were identified with confounders and palindromic sequences). Finally, 28 SNPs were detected for overall LC, 32 for LUAD, 21 for LUSC, and 17 for SCLC (Table S1).

In the reverse MR analysis from UCs to LC, 17 RCC, 17 BCa, and 128 PCa SNPs were selected as IVs based on the following criteria: independence from LD, a genome‐wide significance threshold of *p* < 5 × 10^−6^, and MAF > 0.01. All selected SNPs had an *F* statistic greater than 10. The study also omitted IVs associated with confounders (smoking and BMI) and palindromic sequences (7 RCC, 2 BCa, 40 PCa SNPs were identified as confounders and palindromic sequences). Lastly, 10 SNPs were detected for RCC, 15 for BCa, and 88 for PCa (Table S1).

### 3.2 MR Results of LC to UCs

**FIGURE 2:**
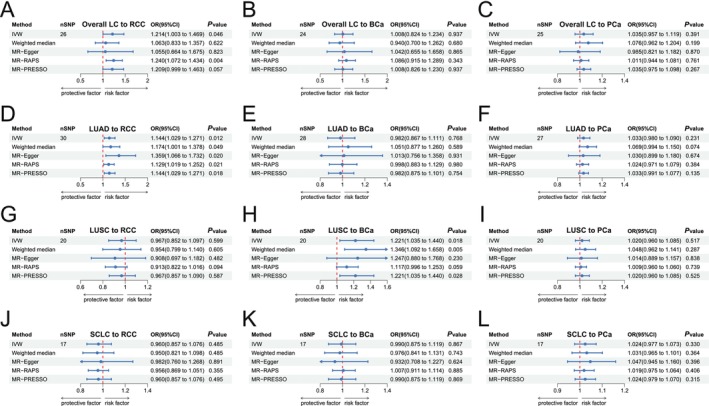
Forest plot of two‐sample Mendelian randomization (MR) study based on the MR method from LC to UCs. (A–C) MR estimates of genetically predicted overall LC on RCC (A), BCa (B) and PCa (C) risk. (D–F) MR estimates of genetically predicted LUAD on RCC (D), BCa (E) and PCa (F) risk. (G–I) MR estimates of genetically predicted LUSC on RCC (G), BCa (H) and PCa (I) risk. (J–L) MR estimates of genetically predicted SCLC on RCC (J), BCa (K) and PCa (L) risk. BCa, bladder cancer; CI, confidence interval; IVW, inverse variance weighted; LC, lung cancer; LUAD, lung adenocarcinoma; LUSC, squamous cell lung carcinoma; MR‐PRESSO, Mendelian randomization pleiotropy residual sum and outlier; MR‐RAPS, Mendelian randomization robust adjusted profile score; OR, odds ratio; PCa, prostate cancer; RCC, renal cell carcinoma; SCLC, small cell lung cancer; SNP, single nucleotide polymorphism; UCs, urological cancers.

**FIGURE 3:**
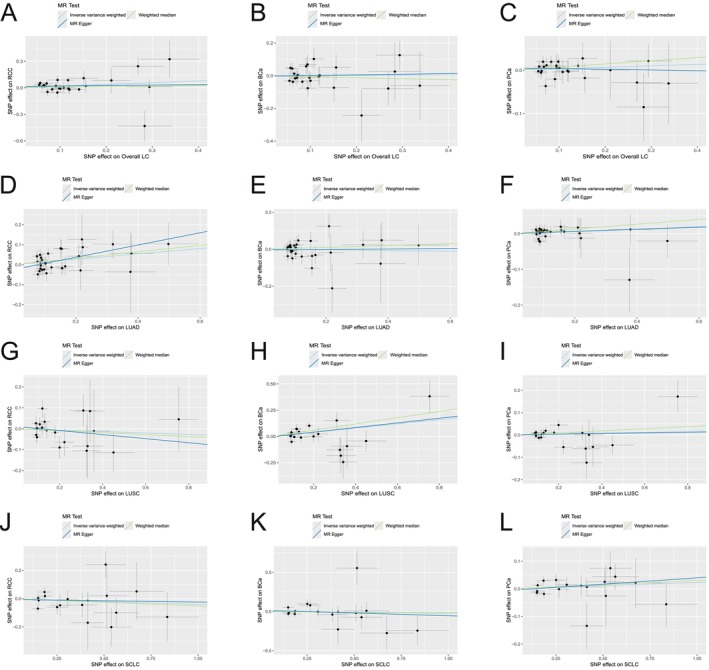
The scatterplots represent genetic IVs association between LC and UCs (forward MR analysis). (A–C) Plots of the effect size of each SNP of overall LC on RCC (A), BCa (B) and PCa (C) risk. (D–F) Plots of the effect size of each SNP of LUAD on RCC (D), BCa (E) and PCa (F) risk. (G–I) Plots of the effect size of each SNP of LUSC on RCC (G), BCa (H) and PCa (I) risk. (J–L) Plots of the effect size of each SNP of SCLC on RCC (J), BCa (K) and PCa (L) risk. BCa, bladder cancer; LC, lung cancer; LUAD, lung adenocarcinoma; LUSC, squamous cell lung carcinoma; PCa, prostate cancer; RCC, renal cell carcinoma; SCLC, small cell lung cancer; SNP, single nucleotide polymorphism; UCs, urological cancers.

In forward‐direction MR, in the overall LC to RCC MR study, IVW revealed credible evidence that overall LC was associated with an increased risk of RCC at the genetic level (IVW: OR = 1.214; 95% CI 1.003–1.469, *p* = 0.046; Figures 2A and 3A). However, no substantial causal association of overall LC to BCa (IVW: OR = 1.008; 95% CI 0.824–1.234; *p* = 0.937; Figures 2B and 3B) and overall LC to PCa (IVW: OR = 1.035; 95% CI 0.957–1.119; *p* = 0.391; Figures 2C and 3C) was found. In the subgroup analysis of overall LC pathological subtypes, LUAD raised the risk of RCC (IVW: OR = 1.144; 95% CI 1.029–1.271, *p* = 0.012; Figures 2D and 3D) but was not correlated with BCa (IVW: OR = 0.982; 95% CI 0.867–1.111; *p* = 0.768; Figures 2E and 3E) and PCa (IVW: OR = 1.033; 95% CI 0.980–1.090; *p* = 0.231; Figures 2F and 3F). LUSC was correlated with the higher risk of BCa (IVW: OR = 1.221; 95% CI 1.035–1.440; *p* = 0.018; Figures 2H and 3H), while there was no causal association with RCC (IVW: OR = 0.967; 95% CI 0.852–1.097; *p* = 0.599; Figures 2G and 3G) and PCa (IVW: OR = 1.020; 95% CI 0.960–1.085; *p* = 0.517; Figures 2I and 3I). As for SCLC, the study also failed to observe a substantial causal relationship with RCC (IVW: OR = 0.960; 95% CI 0.857–1.076; *p* = 0.485; Figures 2J and 3J), BCa (IVW: OR = 0.990; 95% CI 0.875–1.119; *p* = 0.867; Figures 2K and 3K) and PCa (IVW: OR = 1.024; 95% CI 0.977–1.073; *p* = 0.330; Figures 2L and 3L).

### 3.3 MR Results of UCs to LC

To evaluate the potential for reverse causality between UCs and LC, UCs were considered the exposure, and the overall LC and its subtypes were regarded as the outcome. After screening, the study noted that only RCC and SCLC were genetically associated (IVW: OR = 1.185; 95% CI 1.018–1.378; *p* = 0.028; Figures S1D and S2D). Moreover, no reverse causal relationship was found for the other conditions (Figures S1 and S2).

### 3.4 Horizontal Pleiotropy and Heterogeneity Test

**FIGURE 4:**
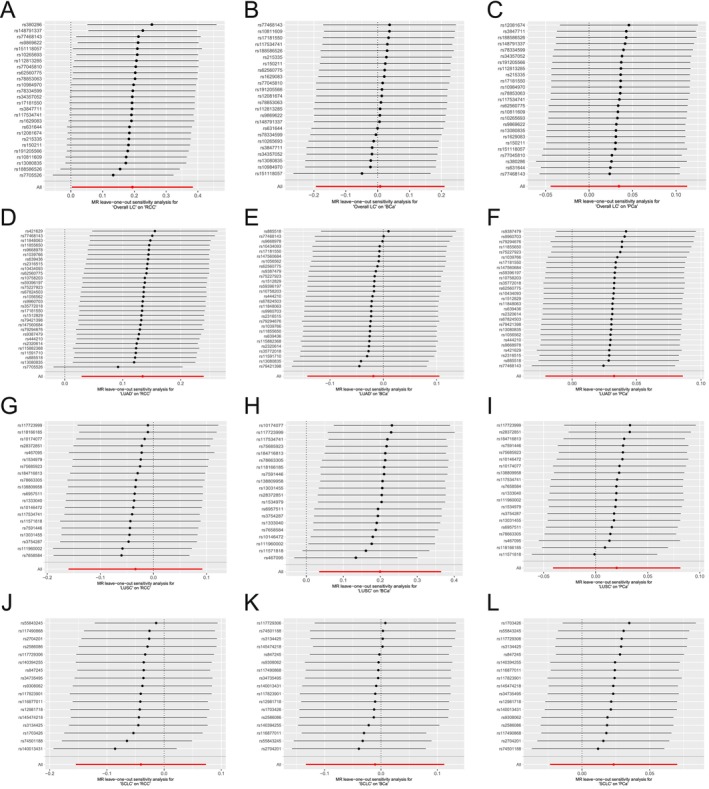
The leave‐one‐out sensitivity analysis for LC on UCs. (A–C) Plots of each SNP of overall LC on RCC (A), BCa (B) and PCa (C) risk. (D–F) Plots of each SNP of LUAD on RCC (D), BCa (E) and PCa (F) risk. (G–I) Plots of each SNP of LUSC on RCC (G), BCa (H) and PCa (I) risk. (J–L) Plots of each SNP of SCLC on RCC (J), BCa (K) and PCa (L) risk. BCa, bladder cancer; LC, lung cancer; LUAD, lung adenocarcinoma; LUSC, squamous cell lung carcinoma; MR, Mendelian randomization; PCa prostate cancer; RCC, renal cell carcinoma; SCLC, small cell lung cancer; UCs, urological cancers.

The heterogeneity and horizontal pleiotropy among genetic instruments were presented in Table S2. In LC to UCs MR analysis, Cochrane's *Q* tests revealed no heterogeneity between LC and UCs IVs (all *p* > 0.05). The genetic association of LC to UCs MR was not driven by a single SNP, as indicated by the leave‐one‐out plot (Figure 4). However, in PCa to LC MR analysis, Cochrane's *Q* tests depicted that some heterogeneity was found between them (overall LC: *Q* = 114.049, *p* = 0.004; LUAD: *Q* = 103.437, *p* = 0.011; LUSC: *Q* = 96.940, *p* = 0.038; LUAD: *Q* = 93.935, *p* = 0.042; Table S2). The genetic association in PCa to LC MR was not driven by a single SNP, as indicated by the leave‐one‐out plot (Figure S3). In the other MR analysis group, no horizontal pleiotropy was observed.

The results indicated that the MR‐Egger regression failed to find any pleiotropic effects for any MR analyses (all *p* > 0.05). The MR‐PRESSO method identified outliers in part of the MR analysis, and the MR‐PRESSO global test still detected horizontal pleiotropy in PCa to partial LC MR analysis after removing outliers (overall LC: Global Test = 116.658, *p* = 0.005; LUAD: Global Test = 105.418, *p* = 0.010; LUSC: Global Test = 99.942, *p* = 0.041). In the other MR analysis group, no horizontal pleiotropy was observed.

### 3.5 GRS Analysis Results

#### 3.5.1 GRS_LC_ to UCs

Consistent with the MR results, GRS_overall LC_ showed the correlation between overall LC and RCC at the genetic level (OR = 1.229, 95% CI 1.067–1.414, *p* = 0.004) (Table 1). Similarly, GRS_LUAD_ showed the association between LUAD and RCC (OR = 1.125, 95% CI 1.019–1.243, *p* = 0.020) (Table 1). In contrast to the MR results, GRS_LUSC_ did not show any correlation between LUSC and BCa (OR = 1.109, 95% CI 0.992–1.240, *p* = 0.069) (Table 1). The GRS results of the other groups were consistent with the MR analysis and were not associated at the genetic level (Table 1).

**TABLE 1:** The effects of the GRS_LC_ on UCs and the GRS_UCs_ on LC.

Exposure	Outcome	OR (95% CI)	*p* value
Overall LC	RCC	1.229 (1.067–1.414)	0.004*
	BCa	1.083 (0.918–1.276)	0.346
	PCa	1.010 (0.945–1.079)	0.771
LUAD	RCC	1.125 (1.019–1.243)	0.020*
	BCa	0.998 (0.887–1.124)	0.980
	PCa	1.023 (0.972–1.076)	0.382
LUSC	RCC	0.922 (0.829–1.024)	0.130
	BCa	1.109 (0.992–1.240)	0.069
	PCa	1.008 (0.961–1.058)	0.745
SCLC	RCC	0.959 (0.875–1.051)	0.369
	BCa	1.007 (0.914–1.109)	0.885
	PCa	1.018 (0.976–1.061)	0.414
RCC	Overall LC	1.061 (1.011–1.113)	0.015*
	LUAD	1.034 (0.968–1.104)	0.326
	LUSC	1.044 (0.963–1.132)	0.298
	SCLC	1.181 (1.026–1.359)	0.021*
BCa	Overall LC	1.015 (0.974–1.057)	0.482
	LUAD	1.029 (0.973–1.089)	0.313
	LUSC	1.052 (0.987–1.120)	0.118
	SCLC	1.025 (0.925–1.136)	0.640
PCa	Overall LC	1.003 (0.982–1.025)	0.778
	LUAD	0.999 (0.970–1.029)	0.960
	LUSC	1.002 (0.968–1.038)	0.899
	SCLC	1.027 (0.973–1.084)	0.329

Abbreviations: BCa, bladder cancer; CI, confidence interval; GRS, genetic risk score; LC, lung cancer; LUAD, lung adenocarcinoma; LUSC, squamous cell lung carcinoma; OR, odds ratio; PCa, prostate cancer; RCC, renal cell carcinoma; SCLC, small cell lung cancer; UCs, urological cancers.

* *p* value < 0.05.

#### 3.5.2 GRS_UCs_ to LC

For the GRS_UCs_ to LC analysis, in line with the MR results, GRS_RCC_ demonstrates a genetic association between RCC and SCLC (OR = 1.181, 95% CI 1.026–1.359, *p* = 0.021) (Table 1). However, in contrast to the MR results, GRS_RCC_ found an association between RCC and overall LC (OR = 1.061, 95% CI 1.011–1.113, *p* = 0.015) (Table 1). The GRS results for the other groups aligned with the MR analysis, showing no genetic‐level associations (Table 1).

### 3.6 SEER Database Validation

Given the above MR and GRS analysis results, overall LC was related to the higher risk of RCC at the genetic level. This study performed a subgroup analysis of overall LC and found that LUAD was primarily responsible for the increased risk of RCC. Therefore, we further used the SEER database to verify the relationship between LUAD and RCC. The findings revealed that the possibility of developing a second primary RCC (SPC‐RCC) after detection of primary LUAD (PLUAD) was markedly elevated compared to a reference US population with comparable age and racial demographics (SIR = 1.25, 95% CI = 1.02–1.51, AER = 1.08, *p* < 0.05).

Table 2 demonstrates the standardized incidence ratios (SIRs) and absolute excess risks (AERs) of SPC‐RCC after various latencies from a diagnosis of PLUAD. The incidence of SPC‐RCC during the initial period was considerably higher than that of a reference US population of similar age and race distribution within 6–11 months of the diagnosis of PLUAD (SIR = 1.88, 95% CI = 1.15–2.91, AER = 3.57, *p* < 0.05). In contrast to a comparable reference US population, no significant increase occurred within 12–59, 60–119, and 120+ months after PLUAD diagnosis.

**TABLE 2:** Standardized incidence ratios and absolute excess risks of second primary RCC after various latencies from a diagnosis of primary LUAD.

Latency	6–11 months			12–59 months			60–119 months			120+ months		
PYR	26245.59			93634.05			43152.83			34107.74		
Statistics	SIR	CI lower	CI upper	SIR	CI lower	CI upper	SIR	CI lower	CI upper	SIR	CI lower	CI upper
	AER			AER			AER			AER		
Value	1.88*	1.15	2.91	1.24	0.92	1.64	1.21	0.76	1.81	0.90	0.51	1.49
	3.57			1.02			0.91			−0.48		

Abbreviations: AER, absolute excess risk; CI, confidence interval; LUAD, lung adenocarcinoma; PYR, Person years at risk; RCC, renal cell carcinoma; SIR, standardized incidence ratio.

* Significant (*p* value < 0.05) relative risks of developing second primary malignancies in comparison to a standard US population with similar age, gender, and race distributions.

Based on the SEER database, the study further investigated the differences in clinicopathological features between LUAD, RCC, and SPC‐RCC after PLUAD. Following the inclusion and exclusion criteria in methods, approximately 19,396 cases of LUAD, 18,802 cases of RCC, and 19 cases of SPC‐RCC after PLUAD were identified. Table 3 demonstrates the characteristics of patients with or without SPC. The results of the survival analysis showed no difference between RCC and SPC‐RCC after PLUAD, nor between LUAD and SPC‐RCC after PLUAD (Figure S4).

**TABLE 3:** Characteristics of patients with or without SPC in SEER database.

Characteristics	LUAD *N* = 19,396	RCC *N* = 18,802	RCC after LUAD *N* = 19	*p* value (LUAD vs. RCC after LUAD)	*p* value (RCC vs. RCC after LUAD)
Age (years)				0.183	< 0.001
Mean ± SD	67.82 ± 10.84	60.89 ± 13.01	71.32 ± 11.00		
Sex				0.246	1.000
Female	10,237 (52.8)	6921 (36.8)	7 (36.8)		
Male	9159 (47.2)	11,881 (63.2)	12 (63.2)		
Differentiation grade^a^				0.343	0.493
I/II	5381 (27.7)	9377 (49.8)	8 (42.1)		
III/IV	5373 (27.7)	5559 (29.6)	5 (26.3)		
Unknown	8642 (44.6)	3866 (20.6)	6 (31.6)		
Tumor size (mm)				0.854	0.066
Mean ± SD	39.08 ± 22.87	55.11 ± 34.81	44.47 ± 39.38		
T stage				0.918	0.187
T1‐2	11,515 (59.4)	14,283 (76.0)	12 (63.2)		
T3‐4	7881 (40.6)	4519 (24.0)	7 (36.8)		
N stage				< 0.001	0.372
N0	7571 (39.0)	17,529 (93.2)	17 (89.5)		
*N*+^b^	11,825 (61.0)	1273 (6.8)	2 (10.5)		
M stage				< 0.001	0.500
M0	9716 (50.1)	16,388 (87.2)	18 (94.7)		
M1	9680 (49.9)	2414 (12.8)	1 (5.3)		
Chemotherapy				0.001	0.671
Yes	9861 (50.8)	1581 (8.4)	2 (10.5)		
No/unknown	9535 (49.2)	17,221 (91.6)	17 (89.5)		

Abbreviations: LUAD, patients with lung adenocarcinoma only; RCC after LUAD, patients who develop a second primary renal cell carcinoma after primary lung adenocarcinoma; RCC, patients with renal cell carcinoma only; SD, standard deviation; SEER, Surveillance, Epidemiology and End Results; SPC second primary cancer.

^a^ Grade I, well differentiated; grade II, moderately differentiated; grade III, poorly differentiated; grade IV, undifferentiated.

^b^ *N*+ included N1, N2, and N3.

## 4 Discussion

The term SPC denotes the development of a new primary cancer in a patient who previously has been diagnosed with cancer and treated with cancer therapy. Recent inventions in cancer management, screening, and treatments have considerably enhanced the access of patients with early‐stage cancers to effective and timely care, resulting in significant increases in long‐term survival rates. The incidence of SPCs has increased considerably in recent decades as life expectancy has increased and cancer survival durations have extended [28]. Previous research indicates that the occurrence of SPC is markedly elevated in patients with cancer relative to the normal population and is likely to rise with extended survival durations. Genetic predispositions and shared environmental factors could be the main drivers. Previous association studies indicated people with specific types of initial malignancy had an elevated risk of developing another malignancy [29, 30], which is consistent with our findings showing that patients with lung cancer, especially lung adenocarcinoma, are at higher risk of developing renal cell carcinoma. Patients with early‐stage cancer reveal a 1.7‐fold increased risk of SPC development compared to the normal population in the context of primary LC, and almost 13.422% of patients are prone to SPC development [17]. Based on the SEER database, we found that in the case of primary lung adenocarcinoma, the risk of developing second primary RCC was 1.25 times that of the normal healthy population. Given that UCs share multiple risk factors with LC, investigating the correlation between LC and UCs can facilitate the identification of high‐risk patients for early detection and efficient treatment, thereby enhancing patient survival rates.

The exact cause of SPC is still unknown, but observational studies suggest that there may be a correlation between environmental variables, genetic predisposition, and choices of lifestyle in SPC development [31]. Recent observational studies indicate a potential correlation between LC and UCs [7, 32]. On the other hand, owing to various confounding factors and the difficulties inherent in undertaking extensive cohort and case–control investigations, the clinical question of whether and to what extent there is an association between LC and UCs remains to be fully explored. Bidirectional MR provides an efficient and economical solution for addressing these inquiries. Bidirectional MR is conducted using two independent sets of genetic variants related to the exposure and outcome separately, and then performing MR analyses to evaluate causality in both directions [33]. We conducted bidirectional MR analyses to assess the causal relationship between LC and UCs, allowing us to evaluate both upstream and downstream effects in disease progression while avoiding reverse causation [34]. Our study reveals a vital understanding of the bidirectional causal associations between LC and UCs among the European population.

In the study, we systematically investigated for the first time the relationship between LC (overall LC, LUAD, LUSC, and SCLC) and UCs (RCC, BCa, and PCa) at the genetic level by a two‐sample MR approach. The findings indicated that both overall LC and LUAD were substantially correlated with RCC, and LUSC was associated with BCa. However, in the reverse MR analysis, we found no association between them. Moreover, a genetic association was identified between RCC and SCLC. Simultaneously, the results of the GRS method revealed a genetic correlation between overall LC and LUAD with RCC, as well as between RCC and both overall LC and SCLC. Therefore, based on the MR analysis and GRS approach findings, this study focused on the significant associations between overall LC and LUAD and the incidence of RCC. Following this, the SEER database was used to validate the correlation between PLUAD and SPC‐RCC. The findings demonstrated that the possibility of developing SPC‐RCC after a PLUAD diagnosis was markedly higher than estimated for the reference US population (SIR = 1.25), particularly within 6–11 months post‐diagnosis of PLUAD. Based on the SEER database, the study further investigated the differences in clinicopathological features between LUAD, RCC, and SPC‐RCC after PLUAD. The survival analysis results indicated no discernible difference between RCC and SPC‐RCC after PLUAD or between LUAD and SPC‐RCC after PLUAD. This implies that patients with PLUAD and associated SPC‐RCC should prioritize RCC treatment, minimizing intervention in PLUAD, and thus may not require intensive treatment. Therefore, it is crucial to provide guidance for clinical prevention and treatment.

In comparison to the general population, individuals who were eligible for LC screening were 1.6 times more likely to be diagnosed with kidney cancer, as demonstrated by the current study. A comprehensive kidney and lung cancer screening program could identify up to 25% of kidney cancers, aligning with our conclusions: patients with PLUAD have a 1.25 times greater risk of developing a SPC‐RCC than the normal healthy population (SIR = 1.25) [35]. A main LC GWAS by McKay et al. in 2017 revealed substantial genetic disparities among LC histological subtypes, indicating unique mechanisms for the development of LUAD, LUSC, and SCLC [13]. Furthermore, several studies have emphasized the same signaling pathways between LUAD and RCC, including the PI3K‐AKT [36, 37] and JAK–STAT pathways [38, 39], suggesting potential overlapping genetic origins and developmental mechanisms for these two cancers. Moreover, Sushi domain containing 2 (SUSD2) is a type I membrane protein with domains characteristic of adhesion molecules. Cheng et al. discovered that *SUSD2* is significantly downregulated in both RCC and LC, and the silencing of *SUSD2* enhanced the proliferation and clonogenesis of RCC and LC cells [40]. Napsin A is an aspartic protease in the lung and kidney epithelial cells. Studies indicate that napsin A expression is primarily restricted to LUAD in lung tumors, but in renal tumors, it is frequently expressed in renal cell carcinomas, especially the papillary and clear cell subtypes. Napsin A, due to its restricted expression, is a valuable marker that improves the identification of LUAD and RCC [41]. The significant correlation between the expression levels of *SUSD2* and napsin A in both LUAD and RCC suggests a shared molecular pathway that could be targeted for therapeutic interventions. Considering the growing prevalence of lung and kidney cancers, identifying these connections is essential for formulating early detection methods and personalized therapeutic approaches. The interaction between both cancers may suggest a shared etiological component, highlighting the significance of cross‐organ screening in patients confirmed with either cancer type. This association promotes further investigation into lung and kidney carcinogenesis mechanisms, possibly improving patient outcomes via more personalized treatment strategies.

There are several advantages in our MR study. Firstly, it is the first MR investigation to systematically assess the genetic association between LC and UCs based on large‐scale GWAS data. Compared to earlier observational studies, MR analysis may substantially reduce potential biases, such as confounders and reverse causality, thereby strengthening causal inference. Secondly, the GWAS datasets of LC and UCs were primarily derived from the populations of European ancestry, substantially reducing the impact of population stratification. Further, the extrapolation of the US population data from the SEER database was consistent with the MR analysis' findings, suggesting that the derived conclusions are reliable. Finally, in addition to employing the IVW method as the main analytical methodology, we utilized the GRS method as a supplementary secondary analysis in combination with the SEER database for further verification. To ensure precision in the results, multiple MR complementary methods were also used, including the weighted median, MR‐Egger, MR‐RAPS, and MR‐PRESSO methods.

However, we would like to acknowledge some limitations in our study. Firstly, this MR study exclusively involved the European population, while the SEER database predominantly reflects the US population, potentially constraining the generalizability of the results to other demographics. Further research should aim to replicate these findings in varied communities, including Asian and African groups, to ensure wider applicability. Secondly, this study was unable to totally eliminate the effect of horizontal pleiotropy, despite the implementation of rigorous measures to find outlier variants and minimize horizontal pleiotropy. The complex and poorly comprehended biological functions of multiple genetic variants may be the source of residual pleiotropy. Thirdly, the statistical power was as high as 99% when the relationship between overall LC and RCC was investigated, whereas it was less than 80% in the study of LUAD and RCC. To verify the findings and ensure adequate statistical power, it is necessary to employ larger sample sizes and more advanced methodologies. Fourthly, the limited number of IVs led to the adoption of a lower *p*‐value threshold to ensure that at least three SNPs were included in the analysis. While the F‐statistic tests showed no evidence of mild instrument bias and the mean F‐statistics remained consistently high, caution is still warranted when interpreting the results. Finally, GWAS has the potential to offer novel insights into the genes implicated in SPC‐RCC after PLUAD. However, detailed mechanism studies are required to gain a more comprehensive understanding of the underlying pathophysiology.

## 5 Conclusions

This study has identified a genetic association between LC and UCs, establishing a crucial foundation for the precise prevention of SPC‐RCC after PLUAD, indicating an urgent need for higher awareness about the occurrence of SPC‐RCC and the prompt implementation of intervention and treatment. Moreover, the findings highlight the need for further research into the biological pathways associated with these cancers. Functional studies exploring the role of shared genetic variants in carcinogenesis could yield new therapeutic targets, especially for comorbid patients with LUAD and RCC. Moreover, targeted therapies that address common genetic pathways may provide novel treatment options for this subgroup of cancer patients.

## Author Contributions

**Hao Zhou:** conceptualization, methodology, writing – original draft, data curation, validation, visualization, software. **Zihao Feng:** software, data curation, visualization, writing – original draft, funding acquisition. **Cunjing Zheng:** funding acquisition, methodology, validation, writing – original draft. **Ling Xia:** methodology, data curation, visualization, writing – original draft. **Ziran Dai:** software, validation, writing – review and editing. **Zheyu Ai:** software, visualization, writing – review and editing. **Zhenwei Li:** software, formal analysis, writing – review and editing. **Kezhi Liu:** methodology, formal analysis, writing – review and editing. **Yinghan Wang:** software, visualization, writing – review and editing. **Ning Su:** software, validation, writing – review and editing. **Zhenhua Chen:** funding acquisition, conceptualization, writing – review and editing, project administration. **Jing Zhang:** conceptualization, investigation, writing – review and editing. **Xiaohan Jin:** conceptualization, writing – review and editing, project administration, supervision, investigation, funding acquisition.

## Ethics Statement

Additional ethical approval was not required as we used published data for re‐analysis.

## Conflicts of Interest

The authors declare no conflicts of interest.

## Supporting information


**Figure S1:** Forest plot of two‐sample Mendelian randomization study based on the MR method from UCs to LC. (A–D) Mendelian randomization estimates of genetically predicted RCC on overall LC (A), LUAD (B), LUSC (C) and SCLC (D) risk. (E–H) Mendelian randomization estimates of genetically predicted BCa on overall LC (E), LUAD (F), LUSC (G) and SCLC (H) risk. (I–L) Mendelian randomization estimates of genetically predicted PCa on overall LC (I), LUAD (J), LUSC (K) and SCLC (L) risk. BCa, bladder cancer; CI, confidence interval; IVW inverse variance weighted; LC, lung cancer; LUAD, lung adenocarcinoma; LUSC, squamous cell lung carcinoma; MR‐PRESSO, Mendelian randomization pleiotropy residual sum and outlier; MR‐RAPS, Mendelian randomization robust adjusted profile score; OR, odds ratio; PCa, prostate cancer; RCC, renal cell carcinoma; SCLC, small cell lung cancer; SNP, single nucleotide polymorphism; UCs, urological cancers.

**Figure S2:** The scatterplots represent genetic IVs association between UCs and LC (reverse MR analysis). (A–D) Plots of the effect size of each SNP of RCC on overall LC (A), LUAD (B), LUSC (C) and SCLC (D) risk. (E–H) Plots of the effect size of each SNP of BCa on overall LC (E), LUAD (F), LUSC (G) and SCLC (H) risk. (I–L) Plots of the effect size of each SNP of PCa on overall LC (I), LUAD (J), LUSC (K) and SCLC (L) risk. BCa, bladder cancer; LC, lung cancer; LUAD, lung adenocarcinoma; LUSC, squamous cell lung carcinoma; PCa, prostate cancer; RCC, renal cell carcinoma; SCLC, small cell lung cancer; SNP, single nucleotide polymorphism; UCs, urological cancers.

**Figure S3:** The leave‐one‐out sensitivity analysis for UCs on LC. (A–D) Plots of each SNP of RCC on overall LC (A), LUAD (B), LUSC (C) and SCLC (D) risk. (E–H) Plots of each SNP of BCa on overall LC (E), LUAD (F), LUSC (G) and SCLC (H) risk. (I–L) Plots of each SNP of PCa on overall LC (I), LUAD (J), LUSC (K) and SCLC (L) risk. BCa, bladder cancer; LC, lung cancer; LUAD, lung adenocarcinoma; LUSC, squamous cell lung carcinoma; MR, Mendelian randomization; PCa, prostate cancer; RCC, renal cell carcinoma; SCLC, small cell lung cancer; UCs, urological cancers.

**Figure S4:** Survival curves of patients with or without SPC based on SEER database. LUAD, patients with lung adenocarcinoma only; RCC after LUAD, patients who develop a second primary renal cell carcinoma after primary lung adenocarcinoma; RCC, patients with renal cell carcinoma only; SEER, Surveillance, Epidemiology and End Results; SPC, second primary cancer.

**Table S1:** Genetic instrumental variables for lung cancer and urological cancers in the Mendelian randomization study.

**Table S2:** Results of heterogeneity and horizontal pleiotropy test.

## Data Availability

The original contributions in this study are provided in the article and supplementary files. For further inquiries, please contact the corresponding author.
