# Supplementary figures and images for: Genetic Association of Primary Lung Cancer With Urological Cancers: A Bidirectional Mendelian Randomization Study and SEER Database Validation
Source: Cancer Med. 2025 Sep 29;14(19):e71272. doi: 10.1002/cam4.71272 (PMC12477798; doi:10.1002/cam4.71272)

A

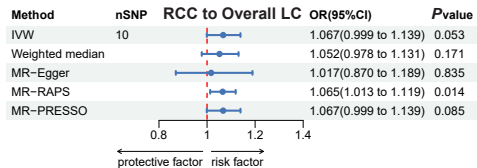

B

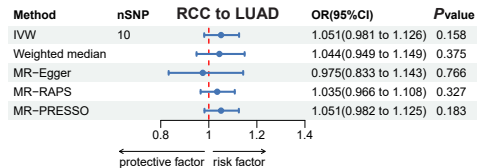

C

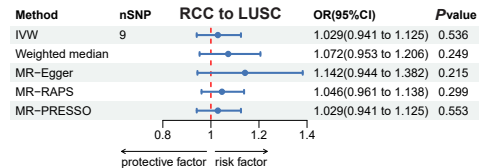

D

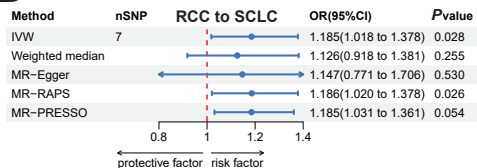

E

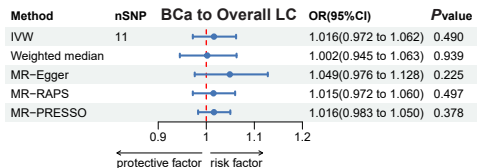

F

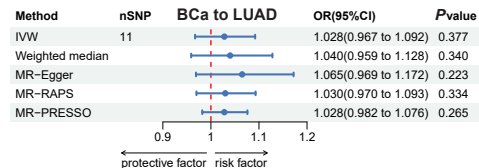

G

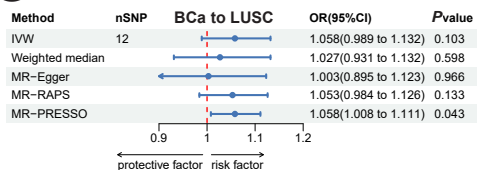

H

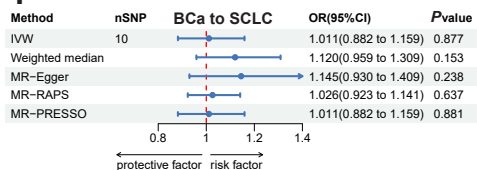

I

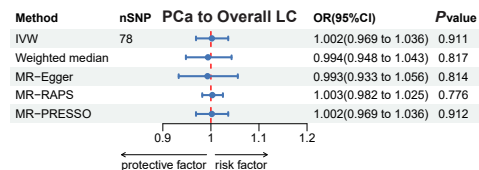

J

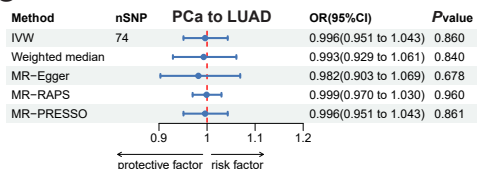

K

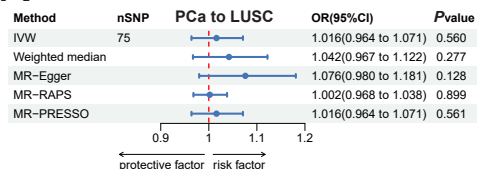

L

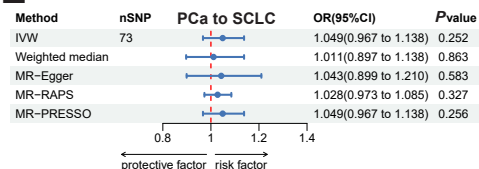

Supplement: Supplementary file 1 — Figure S1: Forest plot of two‐sample Mendelian randomization study based on the MR method from UCs to LC. (A–D) Mendelian randomization estimates of genetically predicted RCC on overall LC (A), LUAD (B), LUSC (C) and SCLC (D) risk. (E–H) Mendelian randomization estimates of genetically predicted BCa on overall LC (E), LUAD (F), LUSC (G) and SCLC (H) risk. (I–L) Mendelian randomization estimates of genetically predicted PCa on overall LC (I), LUAD (J), LUSC (K) and SCLC (L) risk. BCa, bladder cancer; CI, confidence interval; IVW inverse variance weighted; LC, lung cancer; LUAD, lung adenocarcinoma; LUSC, squamous cell lung carcinoma; MR‐PRESSO, Mendelian randomization pleiotropy residual sum and outlier; MR‐RAPS, Mendelian randomization robust adjusted profile score; OR, odds ratio; PCa, prostate cancer; RCC, renal cell carcinoma; SCLC, small cell lung cancer; SNP, single nucleotide polymorphism; UCs, urological cancers. [file CAM4-14-e71272-s004.pdf]

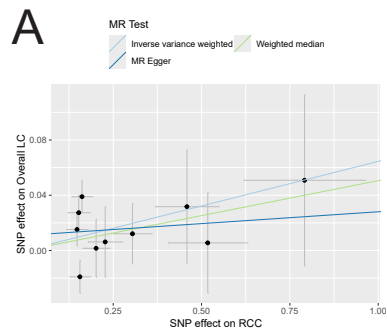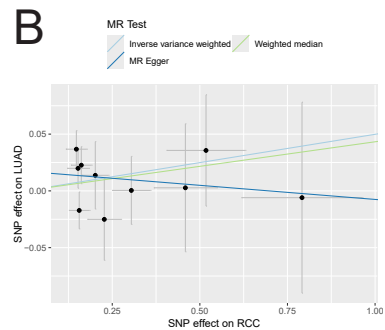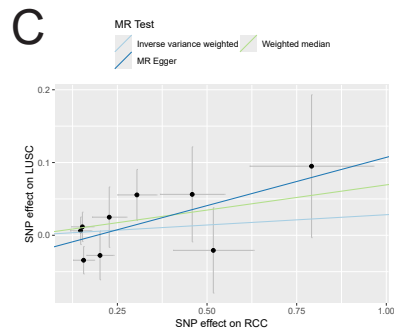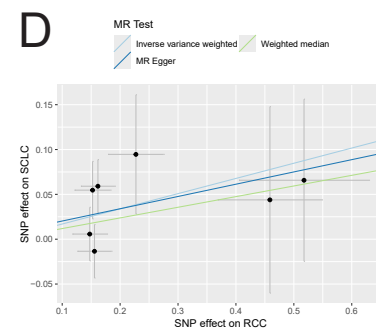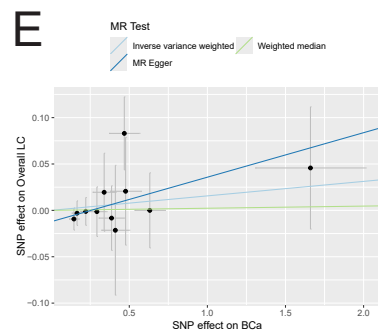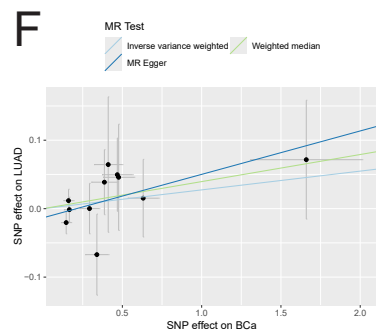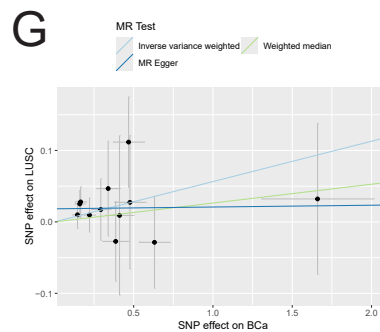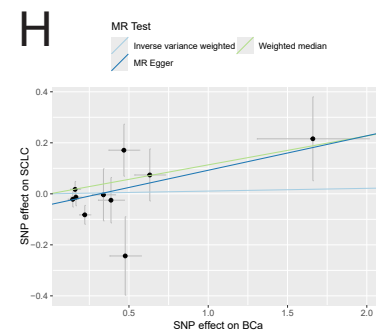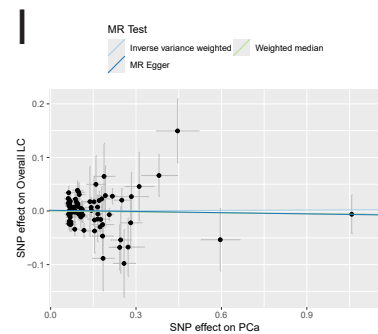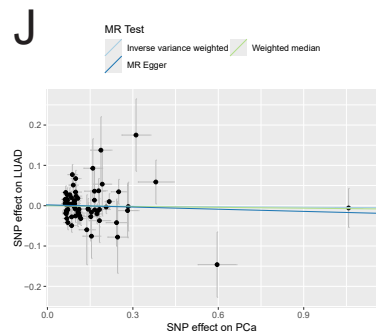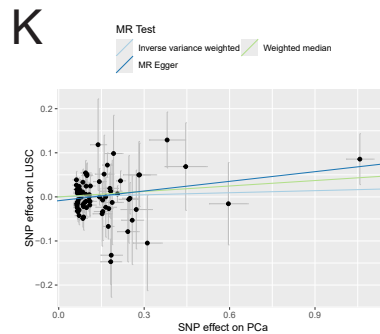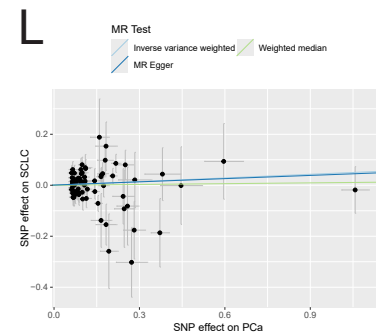

Supplement: Supplementary file 2 — Figure S2: The scatterplots represent genetic IVs association between UCs and LC (reverse MR analysis). (A–D) Plots of the effect size of each SNP of RCC on overall LC (A), LUAD (B), LUSC (C) and SCLC (D) risk. (E–H) Plots of the effect size of each SNP of BCa on overall LC (E), LUAD (F), LUSC (G) and SCLC (H) risk. (I–L) Plots of the effect size of each SNP of PCa on overall LC (I), LUAD (J), LUSC (K) and SCLC (L) risk. BCa, bladder cancer; LC, lung cancer; LUAD, lung adenocarcinoma; LUSC, squamous cell lung carcinoma; PCa, prostate cancer; RCC, renal cell carcinoma; SCLC, small cell lung cancer; SNP, single nucleotide polymorphism; UCs, urological cancers. [file CAM4-14-e71272-s001.pdf]

A

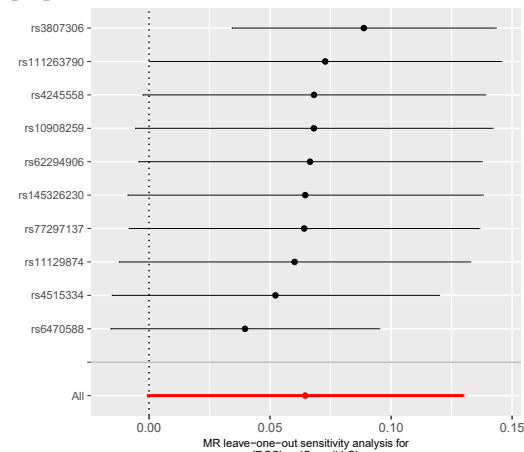

B

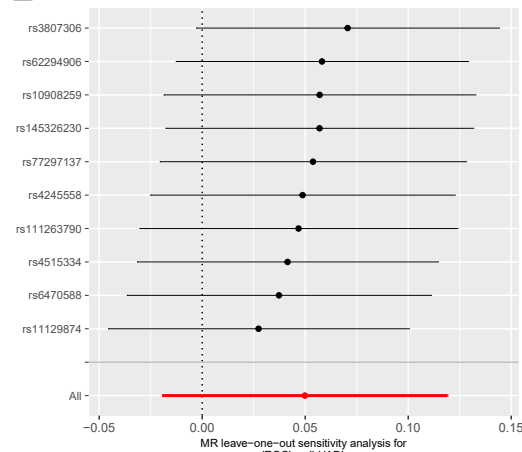

C

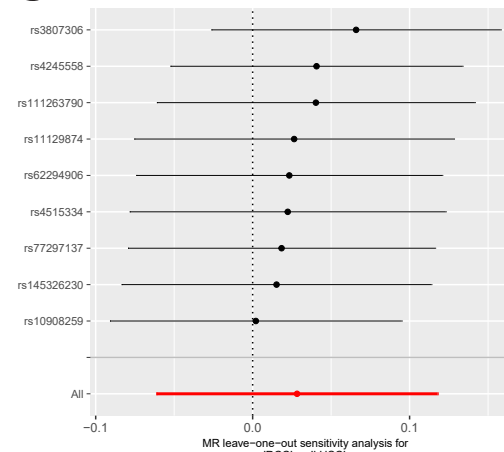

D

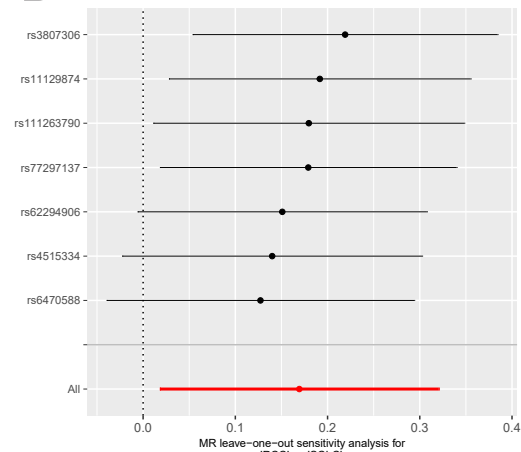

E

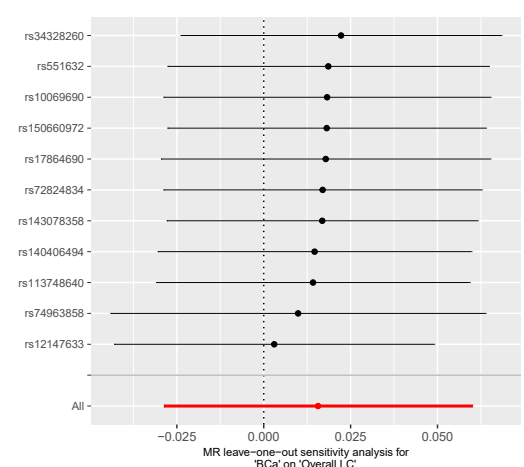

F

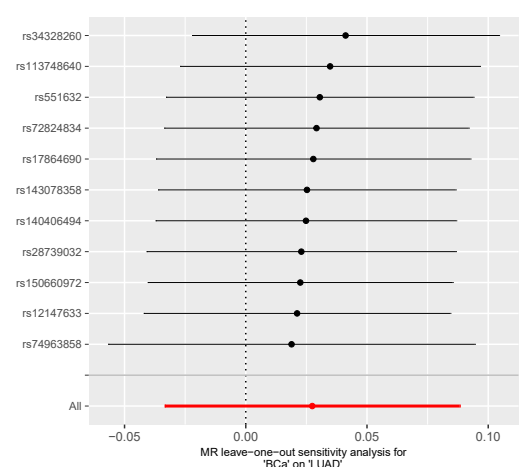

G

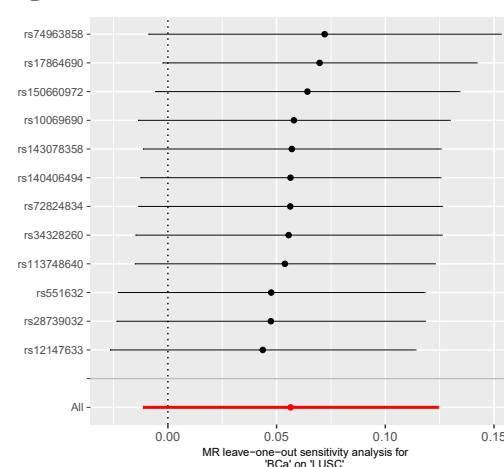

H

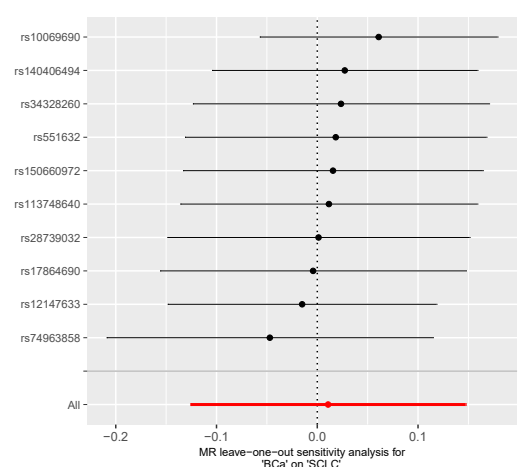

I

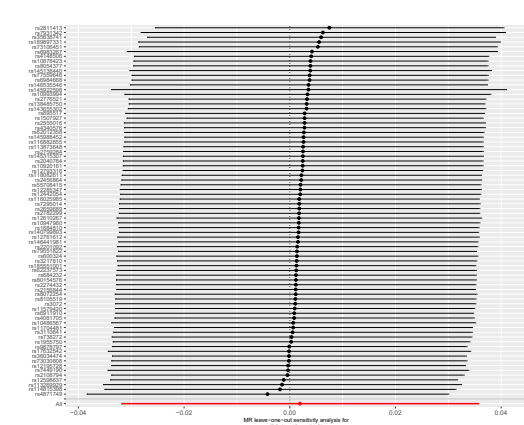

J

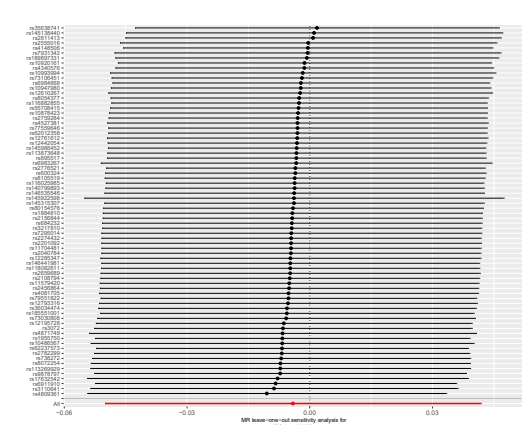

K

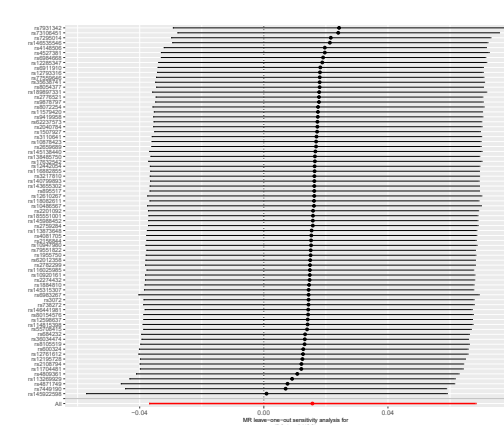

L

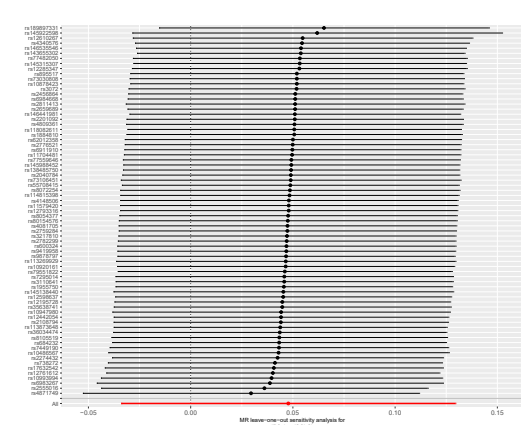

Supplement: Supplementary file 3 — Figure S3: The leave‐one‐out sensitivity analysis for UCs on LC. (A–D) Plots of each SNP of RCC on overall LC (A), LUAD (B), LUSC (C) and SCLC (D) risk. (E–H) Plots of each SNP of BCa on overall LC (E), LUAD (F), LUSC (G) and SCLC (H) risk. (I–L) Plots of each SNP of PCa on overall LC (I), LUAD (J), LUSC (K) and SCLC (L) risk. BCa, bladder cancer; LC, lung cancer; LUAD, lung adenocarcinoma; LUSC, squamous cell lung carcinoma; MR, Mendelian randomization; PCa, prostate cancer; RCC, renal cell carcinoma; SCLC, small cell lung cancer; UCs, urological cancers. [file CAM4-14-e71272-s002.pdf]

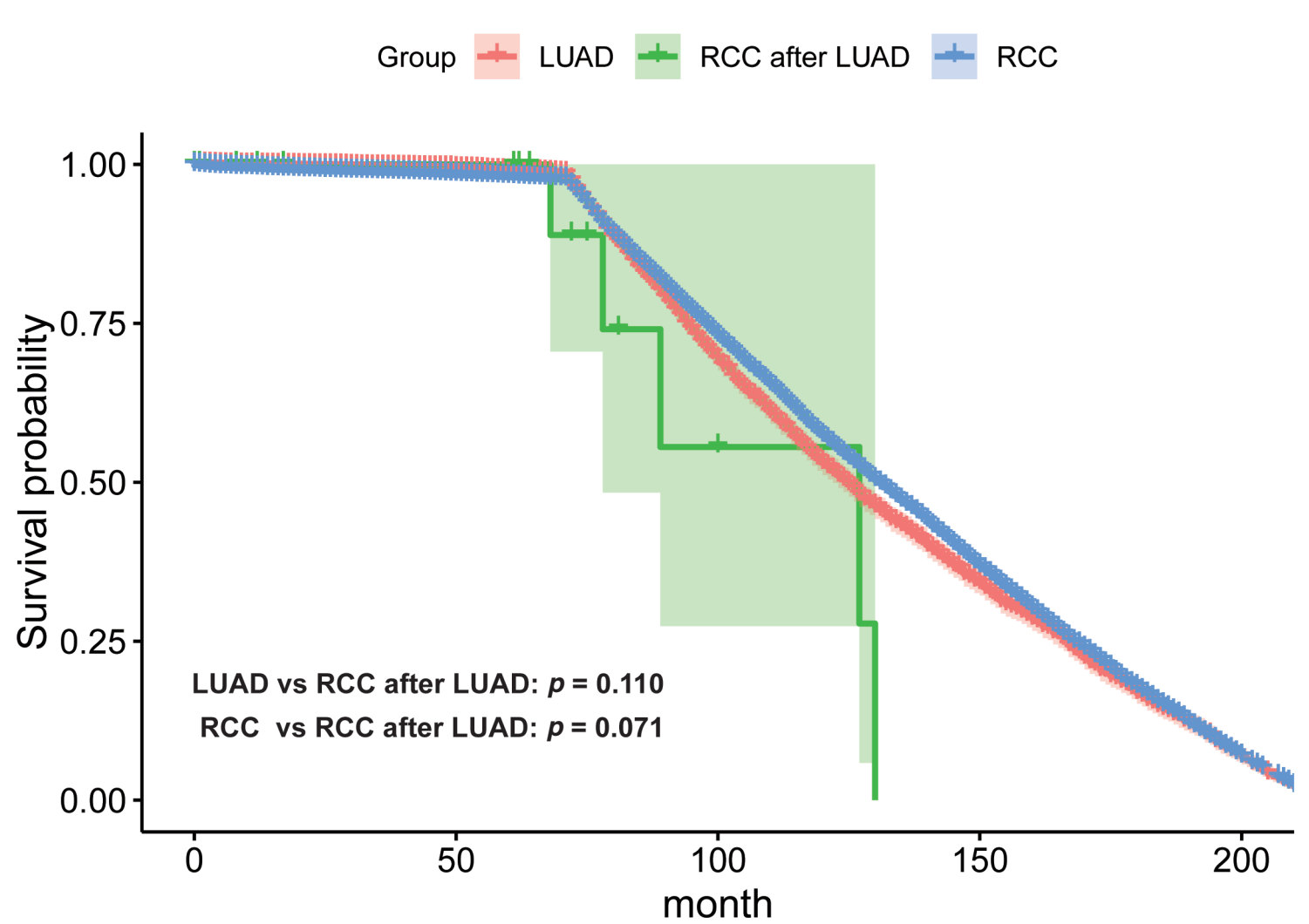

Supplement: Supplementary file 4 — Figure S4: Survival curves of patients with or without SPC based on SEER database. LUAD, patients with lung adenocarcinoma only; RCC after LUAD, patients who develop a second primary renal cell carcinoma after primary lung adenocarcinoma; RCC, patients with renal cell carcinoma only; SEER, Surveillance, Epidemiology and End Results; SPC, second primary cancer. [file CAM4-14-e71272-s005.pdf]
