# Supplementary material for: Genetic Association of Primary Lung Cancer With Urological Cancers: A Bidirectional Mendelian Randomization Study and SEER Database Validation
Source: Cancer Med. 2025 Sep 29;14(19):e71272. doi: 10.1002/cam4.71272 (PMC12477798; doi:10.1002/cam4.71272)
Supplement: Supplementary file 6 — Table S2: Results of heterogeneity and horizontal pleiotropy test. [file CAM4-14-e71272-s006.docx]

| **Supplementary Table 2.** Results of heterogeneity test, horizontal pleiotropy test. | | | | | | | |  |
| --- | --- | --- | --- | --- | --- | --- | --- | --- |
| **Exposure** | **Outcome** | **N_SNP_** | **MR-Egger**  **intercept *P*** | **Global Test** | **Global Test  *P*** | **Cochran’s Q** | **Cochran’s Q *P*** |  |
|  | RCC  BCa  PCa | 26 | 0.519 | 37.414 | 0.12 | 33.830 | 0.112 |  |
| Overall LC |  | 24 | 0.879 | 24.486 | 0.509 | 22.376 | 0.498 |  |
|  |  | 25 | 0.560 | 14.647 | 0.964 | 13.659 | 0.954 |  |
|  | RCC  BCa  PCa | 30 | 0.136 | 33.126 | 0.412 | 30.193 | 0.404 |  |
| LUAD |  | 28 | 0.816 | 25.331 | 0.659 | 23.450 | 0.661 |  |
|  |  | 27 | 0.962 | 16.801 | 0.928 | 15.693 | 0.943 |  |
|  | RCC  BCa  PCa | 20 | 0.602 | 18.971 | 0.606 | 17.225 | 0.575 |  |
| LUSC |  | 20 | 0.891 | 33.886 | 0.054 | 29.577 | 0.057 |  |
|  |  | 20 | 0.917 | 24.258 | 0.280 | 21.581 | 0.306 |  |
|  | RCC  BCa  PCa | 17 | 0.850 | 22.396 | 0.241 | 19.443 | 0.246 |  |
| SCLC |  | 17 | 0.639 | 21.914 | 0.247 | 20.117 | 0.215 |  |
|  |  | 17 | 0.637 | 15.957 | 0.608 | 14.120 | 0.590 |  |
| RCC | Overall LC | 10 | 0.528 | 19.454 | 0.103 | 15.228 | 0.085 |  |
|  | LUAD  LUSC  SCLC | 10 | 0.334 | 10.691 | 0.506 | 8.620 | 0.473 |  |
|  |  | 9 | 0.267 | 11.370 | 0.366 | 8.707 | 0.368 |  |
|  |  | 7 | 0.869 | 6.939 | 0.583 | 5.035 | 0.539 |  |
| BCa | | Overall LC | 11 | 0.295 | 6.611 | 0.863 | 5.615 | 0.846 |
|  |  | LUAD  LUSC  SCLC | 11 | 0.358 | 6.425 | 0.873 | 5.595 | 0.848 |
|  |  |  | 12 | 0.271 | 6.866 | 0.874 | 5.601 | 0.899 |
|  |  |  | 10 | 0.174 | 17.764 | 0.152 | 13.632 | 0.136 |
| PCa | Overall LC  LUAD  LUSC  SCLC | 78 | 0.723 | 116.658 | 0.005^#^ | 114.049 | 0.004^#^ |  |
|  |  | 74  75 | 0.703  0.148 | 105.418  99.942 | 0.010^#^  0.041^#^ | 103.437  96.940 | 0.011^#^  0.038^#^ |  |
|  |  | 73 | 0.928 | 96.812 | 0.051 | 93.935 | 0.042^#^ |  |

^#^: *P* value < 0.05.
